# Amelioration of Cancer Cachexia‐Induced Muscle Atrophy and Adipose Tissue Wasting by a Combination of Amaranth ( *Amaranthus caudatus* L.) Hydrolysate and Korean Mint (*Agastache rugosa*) Extract in BALB/c Mice

**DOI:** 10.1002/fsn3.72182

**Published:** 2026-07-29

**Authors:** Junhee Lee, Junhui Kang, Sungmin Han, Ho‐Tak Jung, Daedong Kim, Dong‐Woo Lee, Jae‐Kwan Hwang

**Affiliations:** ^1^ Graduate Program in Bioindustrial Engineering Yonsei University Seoul Republic of Korea; ^2^ Health Research Division DAESANG Wellife Co., Ltd. Seoul Republic of Korea; ^3^ Department of Biotechnology Yonsei University Seoul Republic of Korea

**Keywords:** adipose tissue wasting, Amaranth ( *Amaranthus caudatus* L.), cancer cachexia, Korean mint (*Agastache rugosa*), muscle atrophy

## Abstract

Cancer cachexia (CC) is a multifactorial metabolic syndrome characterized by systemic inflammation, muscle wasting, and adipose tissue depletion. Amaranth *(Amaranthus caudatus* L.), a pseudocereal rich in proteins, squalene, and tocotrienols, exhibits strong antioxidant and anti‐inflammatory activities. Enzymatic hydrolysis of amaranth proteins enhances bioavailability and physiological potency. Korean mint (*Agastache rugosa*) extract, abundant in flavonoids such as tilianin and acacetin, has been reported to suppress inflammatory and oxidative pathways. However, the combined effect of amaranth hydrolysate and Korean mint extract (AKE) on cancer cachexia has not been elucidated. CT26 tumor‐bearing mice (six‐week‐old male BALB/c) were orally administered AKE (125 or 250 mg/kg/day) for 14 days. AKE attenuated cachexia‐induced weight loss (3.6%–10.3%, *p* < 0.01) and preserved muscle (13.6%–15.8%, *p* < 0.01) and fat mass (36.0%–40.8%, *p* < 0.01). Grip strength was significantly improved. AKE suppressed serum pro‐inflammatory cytokines and inhibited muscle protein degradation by downregulating muscle RING‐finger protein‐1 and muscle atrophy F‐box expression. It also enhanced protein synthesis by activating the protein kinase B/mammalian target of rapamycin pathway. In adipose tissue, AKE reduced AMP‐activated protein kinase browning while promoting adipogenesis via proliferator–activated receptor gamma, CCAAT/enhancer‐binding protein alpha, and Sterol regulatory element‐binding protein 1 upregulation. These findings demonstrate that AKE mitigates cancer‐induced muscle and fat loss by modulating inflammation, proteolysis, and metabolic remodeling, highlighting its potential as a multi‐targeted nutritional strategy for managing cancer cachexia.

## Graphical abstract

Amaranth hydrolysate combined with *Agastache rugosa* extract (AKE) attenuated cancer cachexia in CT26 tumor‐bearing mice by suppressing systemic inflammation and modulating muscle and adipose tissue metabolism. AKE inhibited FoxO3a‐mediated proteolysis while restoring PI3K/Akt/mTOR‐dependent protein synthesis in skeletal muscle. In adipose tissue, AKE reduced AMPK‐mediated browning and promoted adipogenesis by activating PPARγ, C/EBPα, and SREBP1, thereby preserving muscle and fat mass and improving physical function.

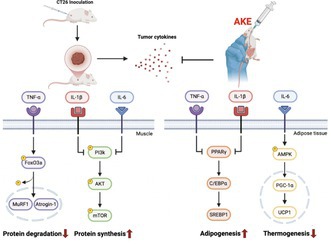

## 1 Introduction

Cancer cachexia (CC) is a multifactorial syndrome characterized by progressive loss of skeletal muscle mass, often accompanied by adipose tissue depletion, leading to reduced quality of life (Argilés et al. 2014). Cachexia compromises functional status, reduces tolerance to anticancer therapies, and increases mortality risk (Porporato 2016). It affects up to 80% of patients with advanced malignancies and is a major contributor to cancer‐related morbidity and mortality (Farkas et al. 2013). Cachexia is particularly prevalent in gastrointestinal cancers, including pancreatic and gastric cancers, as well as in lung and colorectal cancers (Argilés et al. 2014; Fearon et al. 2011). Recent evidence suggests that chronic inflammation and systemic metabolic reprogramming are key drivers of cancer cachexia (Robinson et al. 2023; Wyart et al. 2025).

Mechanistically, cachexia is primarily driven by chronic systemic inflammation, characterized by elevated levels of pro‐inflammatory cytokines such as tumor necrosis factor‐alpha (TNF‐α), interleukin‐6 (IL‐6), and interleukin‐1 beta (IL‐1β), which promote catabolic processes and inhibit anabolic signaling in skeletal muscle and adipose tissue (Onesti and Guttridge 2014; Robinson et al. 2023). In skeletal muscle, these cytokines activate the transcription factor forkhead box O3a (FoxO3a), which induces the upregulation of muscle‐specific E3 ubiquitin ligases, including muscle RING‐finger protein‐1 (MuRF1) and muscle atrophy F‐box protein (atrogin‐1), thereby driving proteasomal degradation of muscle proteins (Webster et al. 2020). Concurrently, inflammatory signaling suppresses the phosphoinositide 3‐kinase (PI3K)/protein kinase B (AKT)/mammalian target of rapamycin (mTOR) pathway, a key regulator of protein synthesis and muscle homeostasis (Webster et al. 2020). In adipose tissue, cytokines activate AMP‐activated protein kinase (AMPK). This kinase promotes the browning of white adipose tissue via the upregulation of thermogenic markers, such as peroxisome proliferator‐activated receptor gamma coactivator 1‐alpha (PGC‐1α) and uncoupling protein 1 (UCP1), enhancing mitochondrial biogenesis and energy expenditure (Daas et al. 2019; Dalal 2019). AMPK also suppresses adipogenesis by downregulating transcription factors, including peroxisome proliferator‐activated receptor gamma (PPARγ), CCAAT/enhancer‐binding protein alpha (C/EBPα), and sterol regulatory element‐binding protein 1 (SREBP1) (Joshi and Patel 2022). Collectively, these processes orchestrate the progressive loss of skeletal muscle and adipose tissue observed in CC.

Because cancer cachexia is driven by multiple interconnected pathological mechanisms, therapeutic strategies targeting a single mechanism have shown limited efficacy. Therefore, multi‐targeted nutritional interventions that simultaneously modulate systemic inflammation, skeletal muscle wasting, and adipose tissue dysfunction have emerged as promising therapeutic strategies for the management of cancer cachexia (Robinson et al. 2023; Van de Worp et al. 2020). Amaranth ( *Amaranthus caudatus* L.), a pseudocereal belonging to the Amaranthaceae family, is native to the Andean region of Central and South America and is widely cultivated there (Schmidt et al. 2023). It is especially recognized for its high‐quality protein, with seeds rich in albumins and globulins and a well‐balanced essential amino acid profile. Notably, *Amaranthus caudatus* L. contains relatively high levels of lysine, an amino acid typically limiting in conventional cereal grains (Martínez‐López et al. 2020). Reported lysine content ranges from approximately 4.8 to 6.7 g per 100 g protein, depending on cultivar and growing conditions, and exceeds that of conventional cereal grains (Martínez‐López et al. 2020). This superior protein composition underlies various physiological benefits, including anticancer, antidiabetic, antihyperlipidemic, antihypertensive, and hypocholesterolemic effects (Rivero Meza et al. 2023; Toimbayeva et al. 2025). Beyond proteins, amaranth seeds contain diverse bioactive compounds, such as squalene, tocotrienols, dietary fiber, phospholipids, and lectins, which further enhance their functional properties (Hong et al. 2014). Collectively, these attributes highlight amaranth as a promising functional food ingredient (Schmidt et al. 2023).

Korean mint (*Agastache rugosa* (Fisch. & C.A.Mey.) Kuntze), a medicinal plant belonging to the Lamiaceae family, has long been used in Korea, Japan and China to treat digestive disorders and inflammatory conditions (Nechita et al. 2023). Recent studies demonstrate that Korean mint extract and its bioactive compound tilianin attenuate muscle atrophy by activating the PI3K/Akt/mTOR pathway and suppressing muscle‐specific ubiquitin ligases in TNF‐α‐treated C2C12 myotubes and immobilization‐induced muscle atrophy models, thereby promoting the synthesis and reducing the degradation of proteins (Woo et al. 2024). These findings indicate that Korean mint exerts muscle hypertrophy effects through coordinated regulation of anabolic and catabolic signaling. Therefore, the complementary nutritional and bioactive properties of amaranth and Korean mint may act synergistically to counteract cancer cachexia by simultaneously targeting multiple pathological mechanisms. This study investigated the therapeutic potential of the combination of amaranth hydrolysate and Korean mint extract (AKE) in a cancer‐induced cachexia model, focusing on the effects of this combination on systemic inflammation, muscle catabolism, and adipose tissue loss.

## 2 Materials and Methods

### 2.1 Preparation of Amaranth Hydrolysate

Amaranth grain powder (DAESANG Wellife Co. Ltd. Seoul, Korea) was enzymatically hydrolyzed using Alcalase (Novozymes, Bagsværd, Denmark) and Flavourzyme (Novozymes) following the method of Ayala‐Niño et al. (2019) with modifications. The specific activities of Alcalase and Flavourzyme were 2.4 Anson units (AU)/g and 500 leucine aminopeptidase units (LAPU)/g, respectively. The powder was dispersed in distilled water (1:5, w/v). Hydrolysis was initiated by adding Alcalase at an enzyme‐to‐substrate (E/S) ratio of 0.3% (w/w), followed by incubation at pH 7 and 50°C for 2 h with constant agitation (140 rpm). Subsequently, Flavourzyme was added at an E/S ratio of 0.1% (w/w) and the reaction was allowed to proceed under the same conditions for an additional 2 h. Enzymatic activity was terminated by heating the mixture in a water bath at 85°C for 10 min. The resulting hydrolysate was lyophilized and milled into a fine powder.

### 2.2 Standardization of the Korean Mint Extract

**FIGURE 1:**
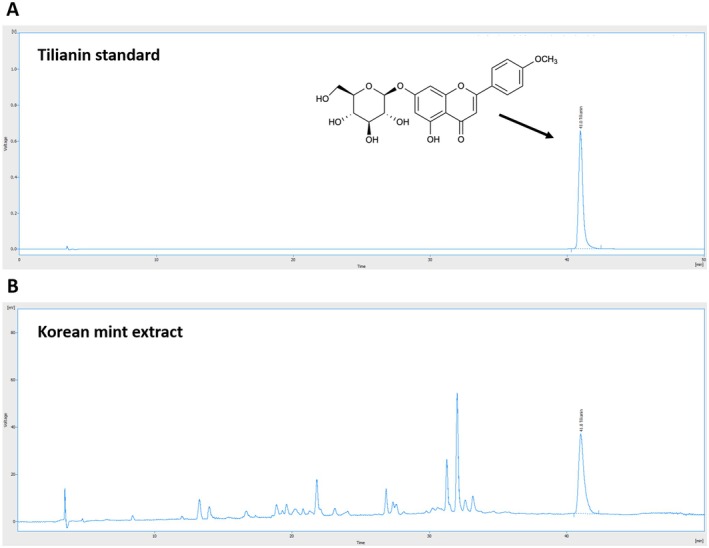
HPLC chromatograms of (A) tilianin and (B) Korean mint extract.

Korean mint extract was obtained from Bolak (Hwasung, Korea). The dried aerial parts of Korean mint were pulverized and extracted with water at 95°C for 4 h. The extract was filtered, concentrated using a rotary vacuum evaporator at 65°C, and spray‐dried with dextrin at an extract‐to‐dextrin ratio of 8:2 (w/w). The yield of the Korean mint extract was approximately 15% (w/w). Standardization was performed using tilianin as the marker compound. Tilianin (> 98% purity; Sigma‐Aldrich, St. Louis, MO, USA) was quantified using a ChroZen high‐performance liquid chromatography (HPLC) system (Youngin Chromass, Anyang, Korea) equipped with a Capcell Pak C18 column (OSAKA SODA, Osaka, Japan). The mobile phase consisted of 0.1% formic acid in water (solvent A) and methanol (solvent B) with the following gradient: 0–30 min, 90%–40% A; 30–40 min, 45% A; 40–45 min, 45%–30% A; and 45–50 min, 30%–90% A. The flow rate was 0.8 mL/min, detection was performed at 280 nm, the column temperature was maintained at 25°C, and the injection volume was 10 μL. HPLC identified tilianin as the major component, with a content of 0.76% (w/w) in the extract (Figure 1).

### 2.3 Preparation of AKE

AKE was prepared by dispersing lyophilized amaranth hydrolysate and Korean mint extract powder separately in saline at a 5:1 (w/w) ratio, followed by thorough mixing. This ratio was selected based on preliminary experiments evaluating anticachectic efficacy using C2C12 myotubes treated with CT26‐conditioned medium (Figure S1). The resulting preparation was used for subsequent experiments.

### 2.4 Cell Culture

CT26 mouse colon carcinoma cells (Korean Cell Line Bank, Seoul, Korea) were cultured in Dulbecco's Modified Eagle Medium (Sigma‐Aldrich) supplemented with 10% fetal bovine serum (HyClone, Logan, UT, USA) and 1% penicillin–streptomycin (100 U/mL penicillin and 100 μg/mL streptomycin) at 37°C in a humidified atmosphere with 5% CO_2_. Prior to injection, the cells were harvested and resuspended in phosphate‐buffered saline (PBS).

### 2.5 Animal Experiments

Six‐week‐old male BALB/c mice were purchased from Samtako Bio Korea (Osan, Korea) and acclimated for 7 days before CT26 cell inoculation. Acclimation was conducted at the Yonsei Laboratory Animal Research Center (Seoul, Korea) under a 12‐h light/12‐h dark cycle, constant temperature (23°C ± 2°C) and relative humidity of 55% ± 5%. Then, the mice were randomly assigned to four groups (*n* = 10 per group): (1) a non‐cachectic control (CON), (2) CC, (3) CC + AKE125 (CC treated with 125 mg/kg/day AKE) and (4) CC + AKE250 (CC treated with 250 mg/kg/day AKE). The right flank of mice was shaved using clippers (Voguers, Bucheon, Korea) and hair removal cream (Nair; Church & Dwight Co. Inc., Princeton, NJ, USA). CC was induced by subcutaneous injection of 1 × 10^6^ CT26 cells into the right flank, whereas mice in the CON group received an equal volume of PBS. Oral administration of AKE (125 or 250 mg/kg/day) was initiated on Day 8 after CT26 cell inoculation and continued for 14 days. Body weight and tumor volume were measured on alternate days. Tumor volume (V) was estimated using electronic calipers (Mitutoyo Co., Kanagawa, Japan) and calculated based on the hemi‐ellipsoid formula: V = (d^2^ × D)/2, where d and D denote the shorter and the longer diameter, respectively (Barés et al. 2021). At the end of the experiment, the mice were euthanized under anesthesia via cardiac puncture. Liver and spleen weights were assessed at the end of the experiment as indicators of systemic toxicity. No significant differences were observed between AKE‐treated and control groups, suggesting that AKE administration did not induce measurable systemic adverse effects (Figure S2). Tumors, skeletal muscles (quadriceps [QD], gastrocnemius [GA], soleus [SOL], tibialis anterior [TA] and extensor digitorum longus [EDL]) and adipose tissues (epididymal white adipose tissue [eWAT], subcutaneous white adipose tissue [sWAT] and brown adipose tissue [BAT]) were extracted. All animal procedures were approved by the Institute of Animal Care and Use Committee (IACUC) of Yonsei University (Seoul, Korea; permit number: IACUC‐202411‐1959‐02).

### 2.6 Grip Strength Test

At the end of the experiment, forelimb and fore/hindlimb grip strengths were assessed using a grip strength meter (Bioseb, Vitrolles, France). The mice were allowed to grasp a wire grid, and the tail was gently pulled backwards until the grip was released. Each parameter was recorded three times per mouse, and mean values were used for subsequent analysis.

### 2.7 Histological Analysis

GA muscle tissue and eWAT were fixed in 10% formalin, embedded in paraffin and sectioned. Tissue sections were stained with hematoxylin and eosin (H&E) and examined under a CK40 inverted microscope (Olympus, Tokyo, Japan) equipped with a T500 camera (eXcope, Daejeon, Korea). The cross‐sectional area (CSA) of muscle fibers and adipocytes was quantified using ImageJ (National Institutes of Health, Bethesda, MD, USA).

### 2.8 Enzyme‐Linked Immunosorbent Assay (ELISA)

Following anesthesia, blood samples were obtained by cardiac puncture and centrifuged at 1500× g at room temperature for 20 min within 1 h of collection to obtain serum. Serum levels of TNF‐α, IL‐6, and IL‐1β were quantified using respective ELISA kits (ABclonal, Woburn, MA, USA) in accordance with the manufacturer's instructions. Absorbance was read at 450 nm using a VersaMax tunable microplate reader (Molecular Devices, Sunnyvale, CA, USA).

### 2.9 Western Blot Analysis

Total protein was extracted from GA muscle tissue and eWAT using NP‐40 lysis buffer (ELPIS Biotech, Daejeon, Korea) supplemented with 0.2% protease inhibitor cocktail (Sigma‐Aldrich). Protein concentration was determined by the Bradford assay. Equal amounts of protein were electrophoretically separated by SDS‐PAGE and transferred to membranes as described previously (Kim et al. 2024). The membranes were incubated overnight at 4°C with primary antibodies against phospho (p)‐FoxO3a, FoxO3a, p‐PI3K, PI3K, p‐Akt, Akt, p‐mTOR, mTOR, p‐AMPK, AMPK, PPARγ, α‐tubulin (Cell Signaling Technology, Beverly, MA, USA), MuRF1, atrogin‐1, PGC‐1α, UCP1, C/EBPα, and SREBP1 (Santa Cruz Biotechnology Inc., CA, USA). After washing, the membranes were incubated with HRP‐conjugated secondary antibodies (Bethyl Laboratories Inc., Montgomery, TX, USA) for 2 h at 4°C. Protein bands were visualized using enhanced chemiluminescence (ECL) solution (Chembio, Hanam, Korea) and captured with a G:BOX EF imaging system (Syngene, Cambridge, UK). Band intensity was quantified using ImageJ (National Institutes of Health).

### 2.10 Statistical Analysis

Statistical analyses were performed using the GraphPad Prism 10 software (GraphPad Software, Boston, MA, USA). Data were presented as mean ± standard deviation (SD). Intergroup differences were assessed for statistical significance using one‐way analysis of variance followed by Tukey's multiple comparison test. A *p*‐value of < 0.05 was considered statistically significant.

## 3 Results

### 3.1 AKE Restored Weight Loss and Muscle Function in CT26‐Bearing Mice

**FIGURE 2:**
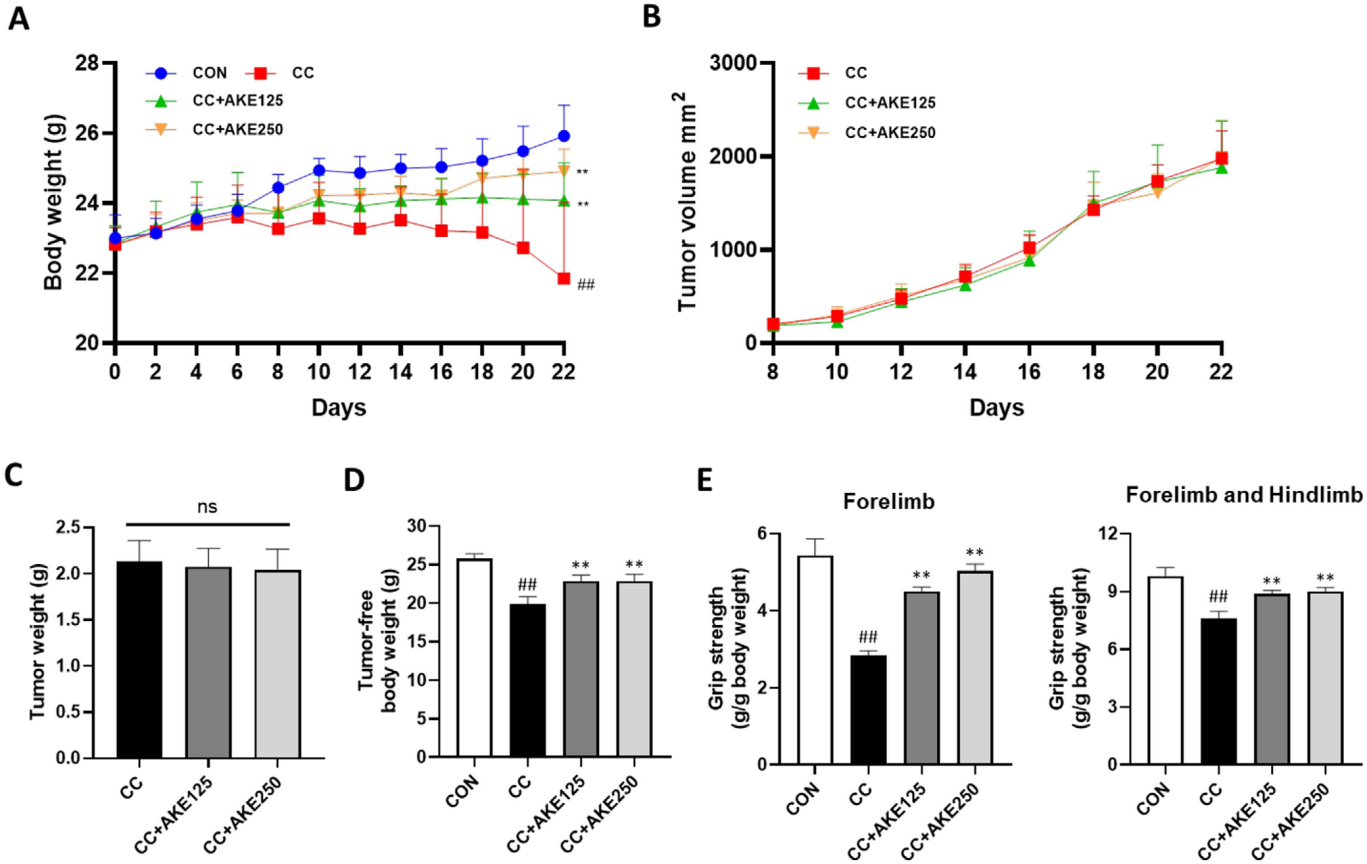
Effects of AKE on body weight and muscle function in CT26‐bearing mice. (A) Body weight was measured every other day. On Day 21 after CT26 inoculation, (B) tumor volume, (C) tumor weight and (D) tumor‐free body weight were recorded. (E) Forelimb and fore/hindlimb grip strengths were measured using a grip strength meter. Data are presented as mean ± standard deviation (SD). *n* = 7 per group. Statistical analysis was performed using one‐way ANOVA followed by Tukey's post hoc test. ^##^ *p* < 0.01 vs. CON group; **p < 0.01 vs. CC group.

Body weight in the CON group steadily increased throughout the experimental period. In contrast, the CC group showed progressive loss from Day 14 and a significant reduction by the end of the study compared to the CON group. AKE administration effectively attenuated cancer‐induced weight loss (Figure 2A). Tumor volume and weight were not affected by AKE treatment (Figure 2B,C). However, body weight, excluding tumor weight, which was reduced in the CC group (Figure 2D), was significantly preserved in AKE‐treated mice. Muscle function was assessed by grip strength. Both forelimb strength and fore/hindlimb strength were markedly reduced in the CC group compared to the CON group. However, the CC + AKE125 and CC + AKE250 groups exhibited an increase in forelimb strength by 58% and 77% and fore/hindlimb strength by 16% and 18%, respectively, compared to the CC group (Figure 2E).

### 3.2 AKE Improved Muscle and Fat Loss in CT26‐Bearing Mice

**FIGURE 3:**
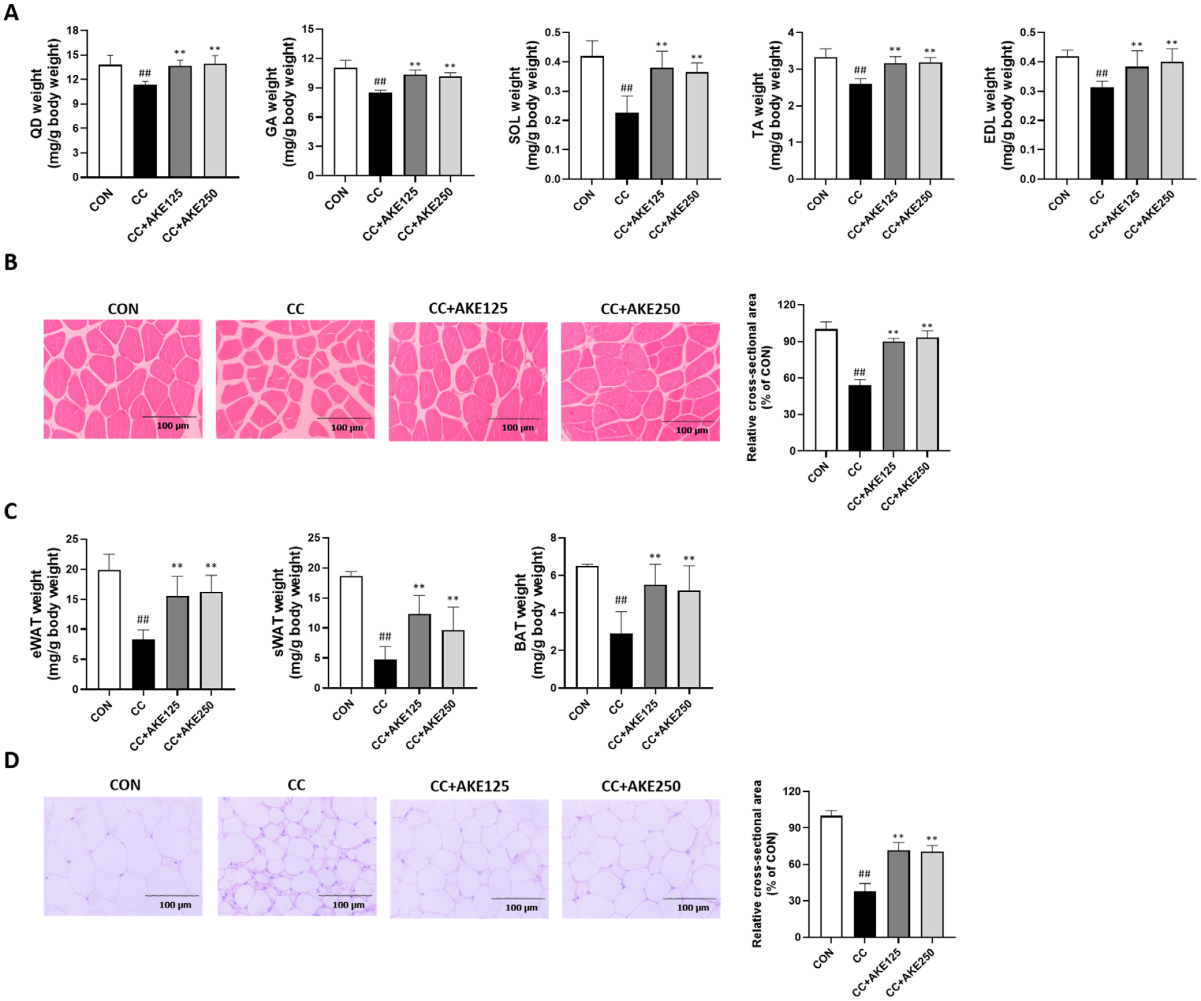
Effects of AKE on muscle and adipose tissue wasting in CT26‐bearing mice. (A) Weights of skeletal muscles (QD, GA, SOL, TA, and EDL) were measured. (B) Cross‐sectional area (CSA) of the GA muscle was evaluated using histological analysis. (C) Weights of adipose tissues (eWAT, sWAT, and BAT) were measured. (D) Adipocyte size in eWAT was evaluated using histological analysis. Data are presented as mean ± standard deviation (SD). *n* = 7 per group. Statistical analysis was performed using one‐way ANOVA followed by Tukey's post hoc test. ##*p* < 0.01 vs. CON group; **p < 0.01 vs. CC group.

Notable reductions in the weights of QD, GA, SOL, TA, and EDL muscles were observed in the CC group, whereas AKE administration significantly preserved muscle mass in CT26‐bearing mice (Figure 3A). The CSA of GA muscle was reduced by 46% in the CC group but was increased by 65% and 71% in the CC + AKE125 and CC + AKE250 groups, respectively, compared to the CON group (Figure 3B). Similarly, the weights of all adipose types (eWAT, sWAT, and BAT) were markedly reduced in the CC group, but restored by AKE treatment in CT26‐bearing mice (Figure 3C). Adipocyte size in eWAT declined by 37% in the CC group but increased by 90% and 87% in the CC + AKE125 and CC + AKE250 groups, respectively, compared to the CC group (Figure 3D).

### 3.3 AKE Suppressed Inflammatory Responses in CT26‐Bearing Mice

**FIGURE 4:**
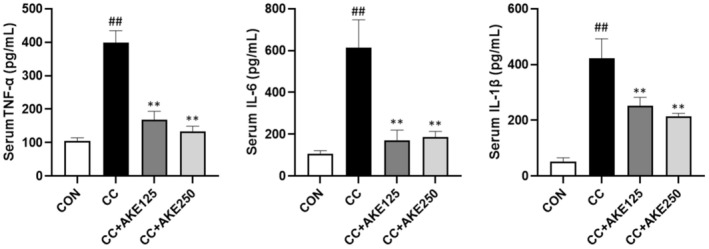
Effects of AKE on inflammatory responses in CT26‐bearing mice. Serum levels of TNF‐α, IL‐6, and IL‐1β were measured by ELISA. Data are presented as mean ± standard deviation (SD). *n* = 5 per group. Statistical analysis was performed using one‐way ANOVA followed by Tukey's post hoc test. ^##^ *p* < 0.01 vs. CON group; **p < 0.01 vs. CC group.

Cachexia is characterized by elevated pro‐inflammatory cytokines, including TNF‐α, IL‐6, and IL‐1β, which drive skeletal muscle and adipose tissue wasting (Onesti and Guttridge 2014; Robinson et al. 2023). In this study, serum levels of TNF‐α, IL‐6, and IL‐1β were higher in the CC group but were markedly reduced in the AKE‐treated groups compared to the CON group (Figure 4).

### 3.4 AKE Attenuated Protein Degradation and Increased Protein Synthesis in CT26‐Bearing Mice

**FIGURE 5:**
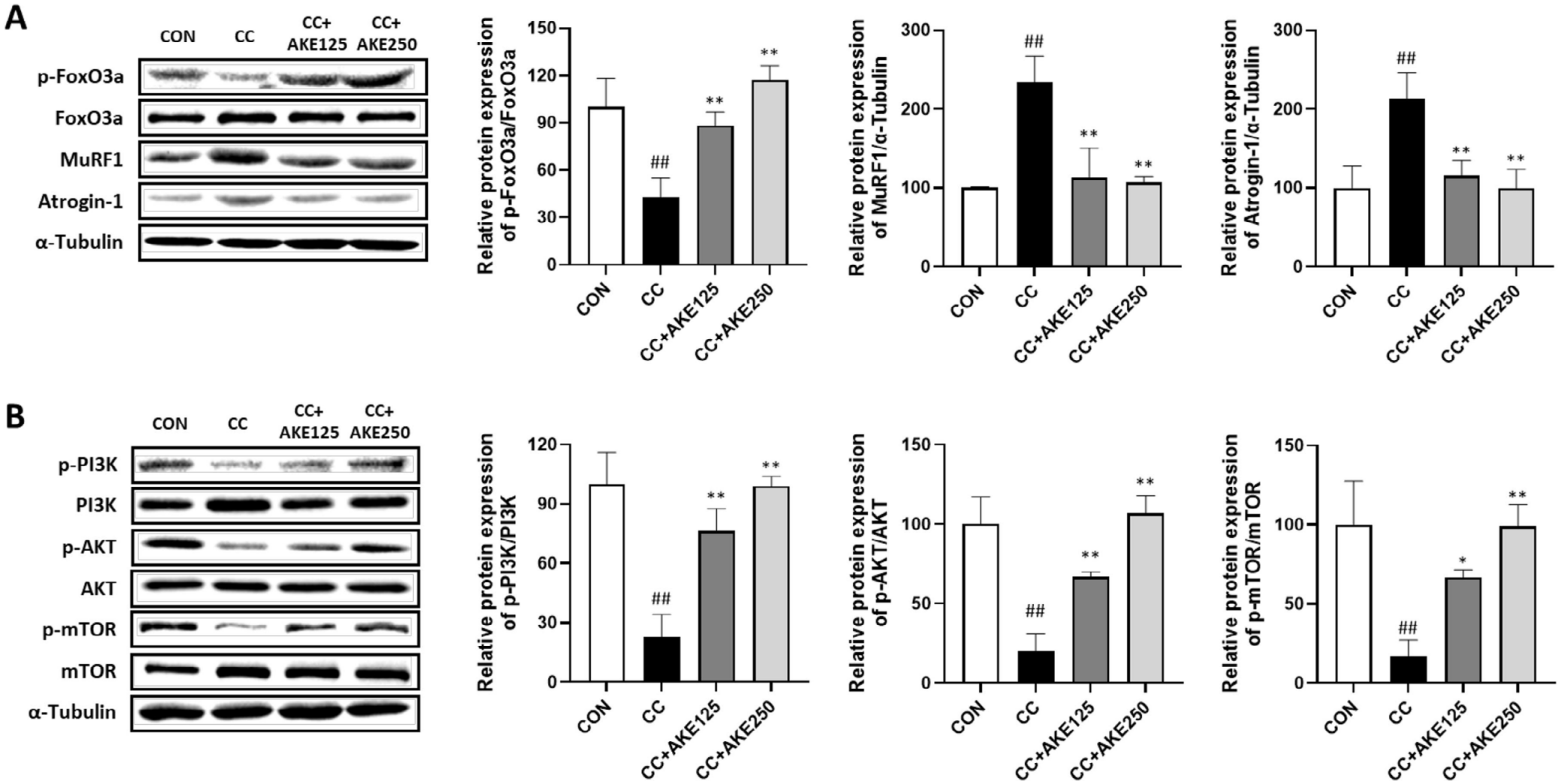
Effects of AKE on muscle protein degradation and synthesis in CT26‐bearing mice. (A) Protein expression of p‐FoxO3a, MuRF1, and atrogin‐1 and (B) protein expression of p‐PI3K, p‐Akt, and p‐mTOR were evaluated by western blotting. α‐Tubulin was used as the loading control. Data are presented as mean ± standard deviation (SD). *n* = 3 per group. Statistical analysis was performed using one‐way ANOVA followed by Tukey's post hoc test. ^##^ *p* < 0.01 vs. CON group; *p < 0.05, **p < 0.01 vs. CC group.

The CC groups exhibited 57% lower p‐FoxO3a expression compared to the CON group, whereas AKE administration restored p‐FoxO3a levels in a dose‐dependent manner, increasing by 108% and 176% in the CC + AKE125 and CC + AKE250 groups, respectively. MuRF1 and atrogin‐1 expressions were significantly upregulated in the CC group compared to the CON group, but markedly suppressed following AKE treatment (Figure 5A). In parallel, the CC group exhibited lower expression of p‐PI3K and p‐Akt compared to the CON group. However, AKE treatment significantly enhanced both markers. Additionally, p‐mTOR expression, which was diminished in the CC group, was dose‐dependently restored by AKE treatment (Figure 5B).

### 3.5 AKE Alleviated WAT Browning and Enhanced Adipogenesis in CT26‐Bearing Mice

**FIGURE 6:**
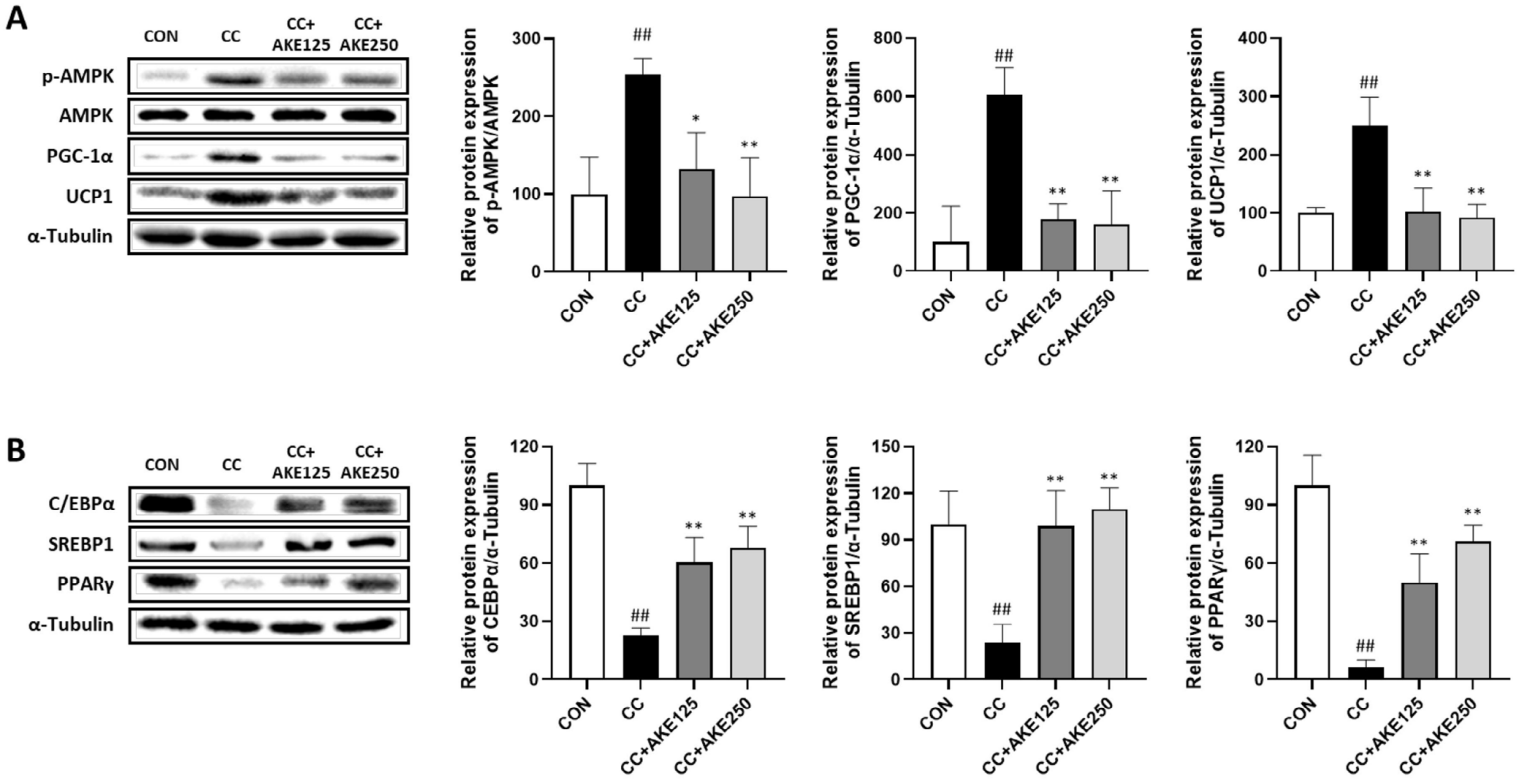
Effects of AKE on adipose tissue browning and adipogenesis in CT26‐bearing mice. (A) Protein expression of p‐AMPK, PGC‐1α and UCP1 and (B) protein expression of C/EBPα, SREBP1 and PPARγ were measured by Western blot. α‐Tubulin was used as the loading control. Data are presented as mean ± standard deviation (SD). *n* = 3 per group. Statistical analysis was performed using one‐way ANOVA followed by Tukey's post hoc test. ^##^ *p* < 0.01 vs. CON group; *p < 0.05, **p < 0.01 vs. CC group.

The CC groups exhibited 154% higher expression of p‐AMPK, a key regulator of WAT browning, compared to the CON group. AKE treatment significantly reduced p‐AMPK expression in a dose‐dependent manner (48% and 62% decrease in the CC + AKE125 and CC + AKE250 groups, respectively). Consistently, downstream thermogenic markers PGC1‐α and UCP1 were upregulated in the CC group but were markedly downregulated by AKE treatment (Figure 6A). In contrast, the CC groups exhibited a downregulation of adipogenic factors C/EBPα, SREBP1, and PPARγ compared to the CON group. However, AKE treatment significantly restored their expression, indicating enhanced adipogenesis (Figure 6B).

## 4 Discussion

CC is a debilitating complication of malignancy and remains one of the most challenging conditions to manage due to its multifactorial pathophysiology and poor response to conventional nutritional and pharmacological therapies (Argilés et al. 2014; Fearon et al. 2011). The syndrome progresses through distinct clinical stages—from pre‐cachexia to refractory cachexia—with therapeutic responsiveness diminishing over time. During the cachexia phase, marked by involuntary weight loss, systemic inflammation, and functional impairment, patients may still derive benefit from targeted interventions. Thus, early implementation of multi‐targeted strategies is essential to preserve muscle and adipose tissue and to mitigate functional decline.

Besides serving as a high‐quality protein source, amaranth hydrolysate appeared to exert distinct bioactivities relevant to cachexia attenuation, suggesting that its effects are mediated not only by protein content but also by specific functional peptides. Previously, amaranth hydrolysate has been shown to exhibit dose‐dependent protective effects in C2C12 myotubes and 3 T3‐L1 adipocytes exposed to CT26‐conditioned media (data not shown). Previous studies have shown that enzymatically hydrolysed amaranth seed proteins possess multifunctional properties, including antihypertensive, antithrombotic, and antioxidant effects, further supporting their therapeutic potential (Ayala‐Niño et al. 2019). Similarly, Korean mint extract, known to promote muscle hypertrophy, has been shown to inhibit the activation of nuclear factor‐kappa B (NF‐κB) and downregulate pro‐inflammatory cytokines (Yin et al. 2024). Given the central role of systemic inflammation in the pathogenesis of CC, these anti‐inflammatory and hypertrophic effects likely contribute to the mitigation of wasting symptoms under cachectic conditions. Taken together, these findings suggest that combining amaranth hydrolysate and Korean mint extract provides a rational, synergistic approach to combat CC, particularly given the need for multi‐targeted interventions.

To evaluate these effects in a physiologically relevant model, CT26 colon carcinoma cells are commonly used to induce cancer cachexia in preclinical models, as they reliably reproduce key features of colorectal cancer‐associated cachexia, including systemic inflammation, body weight loss, and skeletal muscle atrophy (Suzuki et al. 2020). Therefore, the CT26 tumor‐bearing mouse model used in this study provides a well‐established and clinically relevant platform for investigating the effects of AKE on cancer‐associated muscle wasting.

AKE administration significantly reduced body weight loss, preserved muscle and adipose tissue mass, and improved grip strength in CT26‐bearing mice. Histological analyses further demonstrated increased muscle fiber CSA and adipocyte size, indicating structural preservation in both tissues. Similar protective effects have been reported for plant‐derived antioxidants such as curcumin and carnosol, which improved skeletal muscle and adipose tissue integrity in tumor‐bearing models by attenuating inflammation and enhancing metabolic signaling (Ding et al. 2023; Li et al. 2022). Collectively, these findings suggest that AKE not only mitigates functional decline but also preserves muscle and fat architecture, a critical determinant of quality of life in cachectic conditions. The observed phenotypic improvements, therefore, highlight the nutritional potential of AKE as a supportive strategy for CC.

Sustained elevation of pro‐inflammatory cytokines in CC largely originates from tumor‐derived signaling. Tumor cells secrete high levels of TNF‐α, IL‐6, and IL‐1β, which activate catabolic pathways, disrupt metabolic balance, and promote tissue wasting (Onesti and Guttridge 2014). Breakdown of muscle and adipose tissue further aggravates inflammation through the release of damage‐associated molecular patterns (DAMPs), perpetuating the wasting cycle (Robinson et al. 2023). TNF‐α induces muscle proteolysis by activating FoxO3a and its downstream ubiquitin ligases, MuRF1 and atrogin‐1, while also impairing mitochondrial function and protein synthesis (Webster et al. 2020). In adipose tissue, TNF‐α downregulates adipogenic factors and promotes lipolysis, leading to fat depletion (Na et al. 2023). IL‐6 exacerbates muscle wasting by suppressing anabolic signaling and mitochondrial function and promotes adipose browning through AMPK activation and upregulation of PGC‐1α and UCP1 (Daas et al. 2019; VanderVeen et al. 2017). IL‐1β impairs myogenesis and adipogenesis, further disrupting tissue maintenance (Jung et al. 2019; Nagata et al. 2017). In the present study, serum levels of IL‐1β, IL‐6, and TNF‐α were significantly elevated in cachectic mice but were markedly reduced by oral AKE administration. This suppression suggests that AKE mitigates tumor‐derived inflammatory signaling, thereby relieving catabolic pressure on skeletal muscle and adipose tissue. By lowering circulating cytokine levels, AKE potentially contributes to improved protein balance and metabolic homeostasis in these tissues.

In GA muscle tissue, AKE suppressed FoxO3a activation and its downstream targets MuRF1 and atrogin‐1, while enhancing PI3K/AKT/mTOR signaling, indicating coordinated inhibition of catabolic pathways and stimulation of anabolic pathways. Similar regulatory patterns have been described in CC models, where botanical agents, such as *Curcuma xanthorrhiza* and *Dalbergia odorifera*, attenuated muscle wasting by inhibiting FoxO3a activity and restoring PI3K/AKT‐mediated protein synthesis (Ho et al. 2024; Kim et al. 2024). Beyond cancer, soybean protein–derived peptides were shown to mitigate burn injury–induced muscle atrophy via the downregulation of MuRF1 and atrogin‐1, highlighting the shared involvement of cytokine‐driven catabolic signaling in diverse wasting conditions (Zhao et al. 2018).

Under physiological conditions, AMPK activation serves to restore cellular energy homeostasis; however, in the context of CC, sustained cytokine‐driven activation of AMPK leads to maladaptive metabolic remodeling (Dalal 2019). Consistent with this, AMPK expression was significantly increased in the cachexia model. However, AKE administration attenuated AMPK activation and downregulated thermogenic markers in WAT, thereby reversing cachexia‐associated adipose browning. In parallel, AKE enhanced adipogenic signaling by upregulating PPARγ, C/EBPα, and SREBP1, suggesting restoration of lipid storage capacity and adipocyte differentiation. Similar effects were reported with *Arctium lappa* fruit extract in a CC model, where the suppression of AMPK‐driven thermogenesis reduced UCP1 expression, preserved adipocyte structure, and mitigated fat atrophy and body weight loss (Han et al. 2020).

Collectively, these findings demonstrated that AKE exerts coordinated protective effects on skeletal muscle and adipose tissue by modulating key molecular pathways implicated in CC. By suppressing systemic pro‐inflammatory cytokines and restoring tissue‐specific metabolic balance, AKE mitigated muscle and fat degradation while preserving functional capacity and body composition. These findings highlight the potential of AKE as a multi‐targeted nutritional strategy for the management of CC. However, despite the encouraging preclinical outcomes observed in the present study, it remains uncertain whether AKE can achieve comparable therapeutic efficacy in human patients with cancer cachexia. Therefore, further studies, including well‐designed clinical trials, are necessary to validate these findings and determine the translational potential of AKE in clinical settings.

## 5 Conclusion

AKE treatment significantly improved key clinical features of cancer‐induced cachexia. It reduced body weight loss, preserved skeletal muscle and adipose tissue mass, increased muscle fiber and adipocyte CSA and enhanced grip strength, demonstrating functional and structural benefits. At the molecular level, AKE suppressed pro‐inflammatory cytokines and reduced the expression of muscle‐specific ubiquitin ligases, AMPK, and browning markers, while concurrently activating PI3K/AKT/mTOR signaling and upregulating adipogenic factors. Collectively, these findings suggest that AKE exerts a multi‐targeted therapeutic effect by modulating inflammatory and metabolic pathways.

## Author Contributions

**Junhui Kang:** writing – original draft, visualization, formal analysis, data curation, methodology, investigation. **Dong‐Woo Lee:** writing – review and editing, supervision, conceptualization. **Daedong Kim:** writing – review and editing, methodology, investigation, data curation, visualization. **Ho‐Tak Jung:** writing – review and editing, methodology, investigation, formal analysis. **Sungmin Han:** writing – original draft, visualization, formal analysis, data curation, software, investigation. **Junhee Lee:** writing – original draft, visualization, formal analysis, methodology, data curation, investigation. **Jae‐Kwan Hwang:** writing – review and editing, supervision, resources, conceptualization, project administration.

## Funding

This research was supported by a Yonsei scholarship and an Industry–University Cooperative Project funded by DAESANG Wellife Co. Ltd. (2024‐11‐1974).

## Disclosure

No generative artificial intelligence (AI) or AI‐assisted technologies were used in the writing of this manuscript.

## Ethics Statement

Animal experiments were conducted in accordance with protocols approved by the Institutional Animal Care and Use Committee (IACUC) of Yonsei University (permit number: IACUC‐A‐202411‐1959‐02).

## Conflicts of Interest

The authors declare no conflicts of interest.

## Supporting information


**Figure S1:** Determination of the optimal mixing ratio of AKE based on p‐mTOR expression in C2C12 myotubes. (A) Protein expression of p‐mTOR in C2C12 myotubes treated with different mixing ratios of amaranth hydrolysate and Korean mint extract. (B) Protein expression of p‐mTOR in C2C12 myotubes treated with amaranth hydrolysate and Korean mint extract alone or in combination to evaluate potential synergistic effects. Protein levels were determined by Western blot analysis. α‐Tubulin was used as the loading control. Data are presented as mean ± standard deviation (SD). *n* = 3 per group. Statistical analysis was performed using one‐way ANOVA followed by Duncan's multiple range test. ^*##*^ *p* < 0.01 vs. CON; **p < 0.01 vs. CC; ^*$*^ *p* < 0.05, ^*$$*^ *p* < 0.01 vs. each group.
**Figure S2:** Effects of AKE on hepatotoxicity and immunotoxicity. (A) Liver weight and (B) spleen weight. Data are presented as mean ± standard deviation (SD). (*n* = 8 per group). Statistical analysis was performed using one‐way ANOVA followed by Tukey's post hoc test. No significance differences (NS) were observed.

## Data Availability

The data that support the findings of this study are available from the corresponding author upon reasonable request.

## References

[fsn372182-bib-0001] Argilés, J. M. , S. Busquets , B. Stemmler , and F. J. López‐Soriano . 2014. “Cancer Cachexia: Understanding the Molecular Basis.” Nature Reviews Cancer 14, no. 11: 754–762. 10.1038/nrc3829. 25291291

[fsn372182-bib-0002] Ayala‐Niño, A. , G. M. Rodríguez‐Serrano , L. G. González‐Olivares , E. Contreras‐López , P. Regal‐López , and A. Cepeda‐Saez . 2019. “Sequence Identification of Bioactive Peptides From Amaranth Seed Proteins ( *Amaranthus hypochondriacus* Spp.).” Molecules 24, no. 17: 3033. 10.3390/molecules24173033. 31438557 PMC6749583

[fsn372182-bib-0003] Barés, G. , A. Beà , L. Hernández , et al. 2021. “ENDOG Impacts on Tumor Cell Proliferation and Tumor Prognosis in the Context of PI3K/PTEN Pathway Status.” Cancers 13, no. 15: 3803. 10.3390/cancers13153803. 34359707 PMC8345062

[fsn372182-bib-0004] Daas, S. I. , B. R. Rizeq , and G. K. Nasrallah . 2019. “Adipose Tissue Dysfunction in Cancer Cachexia.” Journal of Cellular Physiology 234, no. 1: 13–22. 10.1002/jcp.26811. 30078199

[fsn372182-bib-0005] Dalal, S. 2019. “Lipid Metabolism in Cancer Cachexia.” Annals of Palliative Medicine 8, no. 1: 13–23. 10.21037/APM.2018.10.01. 30525767

[fsn372182-bib-0006] Ding, K. , W. Jiang , J. Zhangwang , Y. Wang , J. Zhang , and M. Lei . 2023. “The Potential of Traditional Herbal Active Ingredients in the Treatment of Sarcopenia Animal Models: Focus on Therapeutic Effects and Mechanisms.” Naunyn‐Schmiedeberg's Archives of Pharmacology 396, no. 12: 3483–3501. 10.1007/s00210-023-02639-7. 37526688

[fsn372182-bib-0007] Farkas, J. , S. von Haehling , K. Kalantar‐Zadeh , J. E. Morley , S. D. Anker , and M. Lainscak . 2013. “Cachexia as a Major Public Health Problem: Frequent, Costly, and Deadly.” Journal of Cachexia, Sarcopenia and Muscle 4, no. 3: 173–178. 10.1007/s13539-013-0105-y. 23539127 PMC3774921

[fsn372182-bib-0008] Fearon, K. , F. Strasser , S. D. Anker , et al. 2011. “Definition and Classification of Cancer Cachexia: An International Consensus.” Lancet Oncology 12, no. 5: 489–495. 10.1016/S1470-2045(10)70218-7. 21296615

[fsn372182-bib-0009] Han, Y.‐H. , J.‐G. Mun , H. D. Jeon , et al. 2020. “The Extract of ( *Arctium lappa* ) L. Fruit (Arctii Fructus) Improves Cancer‐Induced Cachexia by Inhibiting Weight Loss of Skeletal Muscle and Adipose Tissue.” Nutrients 12, no. 10: 3195. 10.3390/nu12103195. 33086629 PMC7603378

[fsn372182-bib-0010] Ho, P. T. , E. Park , Q. X. T. Luong , et al. 2024. “Amelioration of Cancer Cachexia by (*Dalbergia odorifera*) Extract Through AKT Signaling Pathway Regulation.” Nutrients 16, no. 21: 3671. 10.3390/nu16213671. 39519503 PMC11547832

[fsn372182-bib-0011] Hong, S.‐Y. , K.‐S. Cho , Y.‐I. Jin , et al. 2014. “Comparison of Growth Characteristics, Antioxidant Activity and Total Phenolic Contents of Amaranthus Species According to the Different Cultivation Regions and Varieties in South Korea.” Korean Journal of Crop Science 59, no. 1: 16–21. 10.7740/kjcs.2014.59.1.016.

[fsn372182-bib-0012] Joshi, M. , and B. M. Patel . 2022. “The Burning Furnace: Alteration in Lipid Metabolism in Cancer‐Associated Cachexia.” Molecular and Cellular Biochemistry 477, no. 6: 1709–1723. 10.1007/s11010-022-04398-0. 35254613

[fsn372182-bib-0013] Jung, T. W. , T. Park , J. Park , et al. 2019. “Phosphatidylcholine Causes Adipocyte‐Specific Lipolysis and Apoptosis in Adipose and Muscle Tissues.” PLoS One 14, no. 4: e0214760. 10.1371/journal.pone.0214760. 30958839 PMC6453443

[fsn372182-bib-0014] Kim, H. , D.‐W. Lee , and J.‐K. Hwang . 2024. “(Curcuma Xanthorrhiza) Extract and Xanthorrhizol Ameliorate Cancer‐Induced Adipose Wasting in CT26‐Bearing Mice by Regulating Lipid Metabolism and Adipose Tissue Browning.” Integrative Medicine Research 13, no. 1: 101020. 10.1016/j.imr.2023.101020. 38298864 PMC10826318

[fsn372182-bib-0015] Li, W. , K. Swiderski , K. T. Murphy , and G. S. Lynch . 2022. “Role for Plant‐Derived Antioxidants in Attenuating Cancer Cachexia.” Antioxidants 11, no. 2: 183. 10.3390/antiox11020183. 35204066 PMC8868096

[fsn372182-bib-0016] Martínez‐López, A. L. , M. d. C. Millán‐Linares , N. M. Rodríguez‐Martín , F. Millán , and S. Montserrat‐de la Paz . 2020. “Nutraceutical Value of Kiwicha (* Amaranthus caudatus L*.).” Journal of Functional Foods 64: 103735. 10.1016/j.jff.2019.103735.

[fsn372182-bib-0017] Na, H. , Y. Song , and H.‐W. Lee . 2023. “Emphasis on Adipocyte Transformation: Anti‐Inflammatory Agents to Prevent the Development of Cancer‐Associated Adipocytes.” Cancers 15, no. 2: 502. 10.3390/cancers15020502. 36672449 PMC9856688

[fsn372182-bib-0018] Nagata, Y. , T. Kiyono , K. Okamura , et al. 2017. “Interleukin‐1beta (IL‐1β)‐Induced Notch Ligand Jagged1 Suppresses Mitogenic Action of IL‐1β on Human Dystrophic Myogenic Cells.” PLoS One 12, no. 12: e0188821. 10.1371/journal.pone.0188821. 29194448 PMC5711031

[fsn372182-bib-0019] Nechita, M.‐A. , A. Toiu , D. Benedec , et al. 2023. “Agastache Species: A Comprehensive Review on Phytochemical Composition and Therapeutic Properties.” Plants 12, no. 16: 2937. 10.3390/plants12162937. 37631149 PMC10459224

[fsn372182-bib-0020] Onesti, J. K. , and D. C. Guttridge . 2014. “Inflammation Based Regulation of Cancer Cachexia.” BioMed Research International 2014: 1–7. 10.1155/2014/168407. PMC4022077 24877061

[fsn372182-bib-0021] Porporato, P. E. 2016. “Understanding Cachexia as a Cancer Metabolism Syndrome.” Oncogene 5, no. 2: e200. 10.1038/oncsis.2016.3. PMC5154342 26900952

[fsn372182-bib-0022] Rivero Meza, S. L. , A. Hirsch Ramos , L. Cañizares , et al. 2023. “A Review on Amaranth Protein: Composition, Digestibility, Health Benefits and Food Industry Utilisation.” International Journal of Food Science and Technology 58, no. 3: 1564–1574. 10.1111/ijfs.16056.

[fsn372182-bib-0023] Robinson, T. P. , T. Hamidi , B. Counts , et al. 2023. “The Impact of Inflammation and Acute Phase Activation in Cancer Cachexia.” Frontiers in Immunology 14: 1207746. 10.3389/fimmu.2023.1207746. 38022578 PMC10644737

[fsn372182-bib-0025] Schmidt, D. , M. R. Verruma‐Bernardi , V. A. Forti , and M. T. M. R. Borges . 2023. “Quinoa and Amaranth as Functional Foods: A Review.” Food Reviews International 39, no. 4: 2277–2296. 10.1080/87559129.2021.1950175.

[fsn372182-bib-0026] Suzuki, T. , S. Von Haehling , and J. Springer . 2020. “Promising Models for Cancer‐Induced Cachexia Drug Discovery.” Expert Opinion on Drug Discovery 15, no. 5: 627–637. 10.1080/17460441.2020.1724954. 32050816

[fsn372182-bib-0027] Toimbayeva, D. , S. Saduakhasova , S. Kamanova , et al. 2025. “Prospects for the Use of Amaranth Grain in the Production of Functional and Specialized Food Products.” Food 14, no. 9: 1603. 10.3390/foods14091603. PMC12071837 40361686

[fsn372182-bib-0028] Van de Worp, W. R. P. H. , A. M. W. J. Schols , J. Theys , A. van Helvoort , and R. C. J. Langen . 2020. “Nutritional Interventions in Cancer Cachexia: Evidence and Perspectives From Experimental Models.” Frontiers in Nutrition 7: 601329. 10.3389/fnut.2020.601329. 33415123 PMC7783418

[fsn372182-bib-0029] VanderVeen, B. N. , D. K. Fix , and J. A. Carson . 2017. “Disrupted Skeletal Muscle Mitochondrial Dynamics, Mitophagy, and Biogenesis During Cancer Cachexia: A Role for Inflammation.” Oxidative Medicine and Cellular Longevity 2017, no. 1: 3292087. 10.1155/2017/3292087. 28785374 PMC5530417

[fsn372182-bib-0030] Webster, J. M. , L. J. A. P. Kempen , R. S. Hardy , and R. C. J. Langen . 2020. “Inflammation and Skeletal Muscle Wasting During Cachexia.” Frontiers in Physiology 11: 597675. 10.3389/fphys.2020.597675. 33329046 PMC7710765

[fsn372182-bib-0031] Woo, Y. K. , M. Kang , C. Kim , and J.‐K. Hwang . 2024. “Korean Mint (*Agastache rugosa*) Extract and Its Bioactive Compound Tilianin Alleviate Muscle Atrophy via the PI3K/Akt/FoxO3 Pathway in C2C12 Myotubes.” Preventive Nutrition and Food Science 29, no. 2: 154–161. 10.3746/pnf.2024.29.2.154. 38974592 PMC11223928

[fsn372182-bib-0032] Wyart, E. , G. Carrà , E. Angelino , F. Penna , and P. E. Porporato . 2025. “Systemic Metabolic Crosstalk as Driver of Cancer Cachexia.” Trends in Endocrinology and Metabolism 36, no. 9: 815–826. 10.1016/j.tem.2024.12.005. 39757061

[fsn372182-bib-0033] Yin, S. , K. Han , D. Wu , et al. 2024. “Tilianin Suppresses NLRP3 Inflammasome Activation in Myocardial Ischemia/Reperfusion Injury via Inhibition of TLR4/NF‐κB and NEK7/NLRP3.” Frontiers in Pharmacology 15: 1423053. 10.3389/fphar.2024.1423053. 39508038 PMC11538317

[fsn372182-bib-0034] Zhao, F. , Y. Yu , W. Liu , et al. 2018. “Small Molecular Weight Soybean Protein‐Derived Peptides Nutriment Attenuates Rat Burn Injury‐Induced Muscle Atrophy by Modulation of Ubiquitin–Proteasome System and Autophagy Signaling Pathway.” Journal of Agricultural and Food Chemistry 66, no. 11: 2724–2734. 10.1021/acs.jafc.7b05387. 29493231

