# Supplementary material for: Amelioration of Cancer Cachexia‐Induced Muscle Atrophy and Adipose Tissue Wasting by a Combination of Amaranth ( Amaranthus caudatus L.) Hydrolysate and Korean Mint (Agastache rugosa) Extract in BALB/c Mice
Source: Food Sci Nutr. 2026 Jul 29;14(8):e72182. doi: 10.1002/fsn3.72182 (PMC13417478; doi:10.1002/fsn3.72182)
Supplement: Supplementary file 1 — Figure S1: Determination of the optimal mixing ratio of AKE based on p‐mTOR expression in C2C12 myotubes. (A) Protein expression of p‐mTOR in C2C12 myotubes treated with different mixing ratios of amaranth hydrolysate and Korean mint extract. (B) Protein expression of p‐mTOR in C2C12 myotubes treated with amaranth hydrolysate and Korean mint extract alone or in combination to evaluate potential synergistic effects. Protein levels were determined by Western blot analysis. α‐Tubulin was used as the loading control. Data are presented as mean ± standard deviation (SD). n = 3 per group. Statistical analysis was performed using one‐way ANOVA followed by Duncan's multiple range test. ## p < 0.01 vs. CON; **p < 0.01 vs. CC; $ p < 0.05, $$ p < 0.01 vs. each group. Figure S2: Effects of AKE on hepatotoxicity and immunotoxicity. (A) Liver weight and (B) spleen weight. Data are presented as mean ± standard deviation (SD). (n = 8 per group). Statistical analysis was performed using one‐way ANOVA followed by Tukey's post hoc test. No significance differences (NS) were observed. [file FSN3-14-e72182-s001.docx]

**Figure S1**
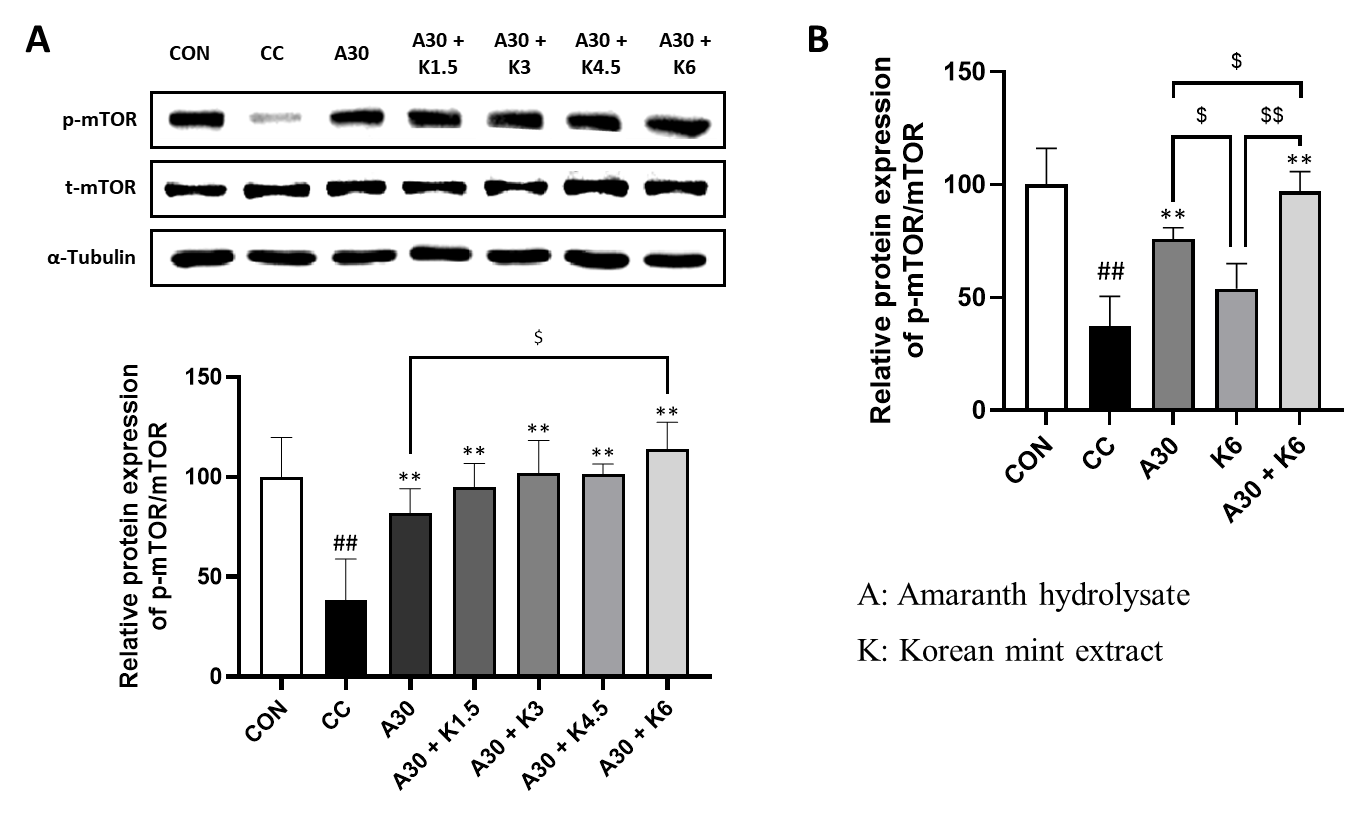


**Figure S1** Determination of the optimal mixing ratio of AKE based on p-mTOR expression in C2C12 myotubes. (A) Protein expression of p-mTOR in C2C12 myotubes treated with different mixing ratios of amaranth hydrolysate and Korean mint extract. (B) Protein expression of p-mTOR in C2C12 myotubes treated with amaranth hydrolysate and Korean mint extract alone or in combination to evaluate potential synergistic effects. Protein levels were determined by Western blot analysis. α-Tubulin was used as the loading control. Data are presented as mean ± standard deviation (SD). n = 3 per group. Statistical analysis was performed using one-way ANOVA followed by Duncan’s multiple range test. *^##^p* < 0.01 vs CON; *^**^p* < 0.01 vs CC; *^$^p* < 0.05, *^$$^p* < 0.01 vs each group.

**Figure S2**

**A B**

**Figure S2** Effects of AKE on hepatotoxicity and immunotoxicity. (A) Liver weight and (B) spleen weight. Data are presented as mean ± standard deviation (SD). (*n* = 8 per group). Statistical analysis was performed using one-way ANOVA followed by Tukey’s post hoc test. No significance differences (NS) were observed.
